# Expression and Role of the G Protein-Coupled Estrogen Receptor (GPR30/GPER) in the Development and Immune Response in Female Reproductive Cancers

**DOI:** 10.3389/fendo.2020.00544

**Published:** 2020-08-20

**Authors:** Christian David Hernández-Silva, Julio César Villegas-Pineda, Ana Laura Pereira-Suárez

**Affiliations:** ^1^Doctorado en Ciencias Biomédicas, Centro Universitario de Ciencias de la Salud, Universidad de Guadalajara, Guadalajara, Mexico; ^2^Laboratorio de Inmunología, Departamento de Fisiología, Centro Universitario de Ciencias de la Salud, Universidad de Guadalajara, Guadalajara, Mexico; ^3^Instituto de Investigación en Ciencias Biomédicas, Centro Universitario de Ciencias de la Salud, Universidad de Guadalajara, Guadalajara, Mexico

**Keywords:** female reproductive cancers, estrogen receptor, G protein-coupled estrogen receptor, GPER, GPR30, estrogen, hormone

## Abstract

Cancer is a major public health issue and represents the second leading cause of death in women worldwide, as female reproductive-related neoplasms are the main cause of incidence and mortality. Female reproductive cancers have a close relationship to estrogens, the principal female sex steroid hormones. Estrogens exert their actions by the nuclear estrogen receptor alpha (ERα) and estrogen receptor beta (ERβ). ERα, and ERβ act as transcription factors mediating genomic effects. Besides, the G protein-coupled estrogen receptor (GPER, formerly known as GPR30) was recently described as a seven-transmembrane receptor that mediates non-genomic estrogenic signaling, including calcium mobilization, cAMP synthesis, cleavage of matrix metalloproteinases, transactivation of epidermal growth factor receptor (EGFR), and the subsequent activation of PI3K and MAPK signaling pathways, which are the reasons why it is related to cellular processes, such as cell-cycle progression, cellular proliferation, differentiation, apoptosis, migration, and invasion. Since its discovery, selective agonists and antagonists have been found and developed. GPER has been implicated in a variety of hormone-responsiveness tumors, such as breast, endometrial, ovarian, cervical, prostate, and testicular cancer as well as lung, hepatic, thyroid, colorectal, and adrenocortical cancers. Nevertheless, GPER actions in cancer are still debatable due to the conflicting information that has been reported to date, since many reports indicate that activation of this receptor can modulate carcinogenesis. In contrast, many others show that its activation inhibits tumor activity. Besides, estrogens play an essential role in the regulation of the immune system, but little information exists about the role of GPER activation on its modulation within cancer context. This review focuses on the role that the stimulation of GPER plays in female reproductive neoplasms, specifically breast, endometrial, ovarian, and cervical cancers, in its tumor activity and immune response regulation.

## Introduction

### Incidence, Mortality, and Prevalence of Gynecological Cancer in the World

Cancer is a leading public health problem in the world (1). It is estimated that incidence and mortality rates will increase by 63.4 and 75.1% by 2040, respectively, due to the growth and aging of the world population (2). Among females, gynecological cancer (including reproductive organs and breast) is the neoplasm with the highest incidence and mortality being the second leading cause of death worldwide (3, 4) (Table 1).

**Table 1:** Incidence, mortality, and prevalence of gynecological cancers (including breast) worldwide in 2018.

**Cancer**	**Incidence (total new cases of all cancers: 8,622,539)**			**Mortality (total deaths of all cancers: 4,169,387)**			**Prevalence, 5 years (total cases of all cancers: 22,826,472)**			**Proportion mortality/incidence**
	**Cancer most frequently diagnosed**	**Number of cases**	**(%)**	**Cause of cancer-related death**	**Number of deaths**	**(%)**	**Most prevalent cancer**	**Number of cases**	**(%)**	
Breast	1st	2,088,849	24.23	1st	626,679	15.03	1st	6,875,099	30.12	0.30
Cervical	4th	569,847	6.61	4th	311,365	7.47	4th	1,474,265	6.46	0.55
Uterine corpus	6th	382,069	4.43	14th	89,929	2.16	5th	1,283,348	5.62	0.24
Ovarian	8th	295 414	3.42	8th	184,799	4.43	7th	762,663	3.34	0.63
Total	N/A	3,336,179	38.69	N/A	1,212,772	29.09	N/A	10,395,375	45.54	N/A

*Information collected from data published at https://gco.iarc.fr/today, accessed [2019 August 19]. N/A, not applicable*.

Breast cancer has the highest incidence, and it is the leading cause of cancer-related death among women worldwide, with 2,088,849 new cases and 626,679 deaths in 2018. Breast cancer represents 30.12% of the prevalent cases of cancer in women in the last 5 years and 15.03% of cancer-related deaths (3, 4).

Cervical cancer is the fourth most incident cancer and the fourth leading cause of cancer-related death worldwide, with 569,847 new cases and 311,365 deaths in 2018. It represents 6.46% of the prevalent cases of cancer in the last 5 years and 7.47% of cancer-related death. In Latin America and the Caribbean, it ranks third in incidence and prevalence rates only after breast and colorectal cancer (4).

In 2018, there were 382,069 new cases of uterine corpus cancer, also known as endometrial cancer, and caused 89,929 deaths, thus being the sixth most frequently diagnosed cancer and fourteenth cancer with more deaths worldwide, representing 5.62% of the prevalent cases of cancer in the last 5 years and 2.16% of cancer-related deaths (4).

In 2018, ovarian cancer was the eighth-most diagnosed cancer with 295,414 new cases and eighth cancer that generated the most deaths, 184,799 to be exact. It is important to mention that, despite having the lowest incidence rate among all gynecological cancers, ovarian cancer reports the highest mortality/incidence ratio (0.63), making it the most lethal (4).

Overall, gynecological cancer had 38.69% of incidence, 29.09% of mortality, and 45.54% of prevalence (5 years) of all cancers in the world in 2018, demonstrating its relevance as a public health problem (4) (Table 1).

### Estrogen and Their Receptors

Estrogen is a steroid hormone associated with the female reproductive organs and is responsible for the development of female sexual characteristics. There are 3 types of natural estrogens: estrone, 17β-estradiol (E2) and estriol. From the previously mentioned forms of estrogen, estradiol is the most common form of estrogenic hormone in the treatment of menopause symptoms as hormone replacement therapy (HRT) (5).

In women, estrogens are synthesized mostly in the ovaries and adrenal glands. Estrogens exert their function primarily through cytosolic estrogen receptors (ERs) existing in target tissues (6). Estrogen is essential for the physiological functions of several organs in the human body. Table 2 summarizes the main effects exerted by this steroid hormone on various organs of the female body.

**Table 2:** Physiological effects of estrogen in various female organs.

**Female organs**	**Physiological effects of estrogen**
Breast	Estrogen is responsible for the development of mammary gland tissue and parenchymal and stromal changes in breast tissue at puberty in females. Estrogen is also responsible for the development of mammary ducts during puberty and, during pregnancy, functions to secrete breast milk in postpartum lactation.
Uterus	In the uterus, estrogen helps to proliferate endometrial cells in the follicular phase of the menstrual cycle, thickening the endometrial lining in preparation for pregnancy.
Vagina	Estrogen supports the proliferation of epithelial mucosa cells of the vagina and the vulva. In the absence of estrogen, the vaginal and vulvar mucosal epithelium becomes thin and presents with symptoms of dryness known as vulvovaginal atrophy.

Canonically, it has been described that estrogen exerts its action at tissues through binding to estrogen receptor alpha (ERα) and estrogen receptor beta (ERβ). Nevertheless, another receptor has recently been described: the G protein-coupled estrogen receptor (GPER, formerly called GPR30) (7, 8).

The genomic estrogen receptors ERα and ERβ belong to the nuclear receptor family and act as transcriptional factors in palindromic sequences called estrogen response elements. They can also interact with other transcriptional factors such as Sp-1, AP-1, and NF-κB to promote the expression of several genes related to cellular processes as proliferation, differentiation, and survival (7, 9).

#### GPER Ligands and Signaling Pathways

Studies show that the three physiological form of estrogen (estrone, E2, and estriol) can bind to GPER. Estrone and E2 have been described as agonists for GPER, while estriol acts as an antagonist. Other molecules that have been found to act as agonists for GPER include therapeutic agents such as diethylstilbestrol, tamoxifen and its metabolite 4-hydroxytamoxifen, ethynylestradiol, raloxifene, and fulvestrant, as well as xenoestrogens such as bisphenol A and phytoestrogens like resveratrol, zearalenone, and genistein (8, 10, 11). Additionally, G-1, a selective agonist (12) and two selective antagonists, G15 (13) and G36 (14), have been synthesized and used in numerous studies.

**Figure 1:**
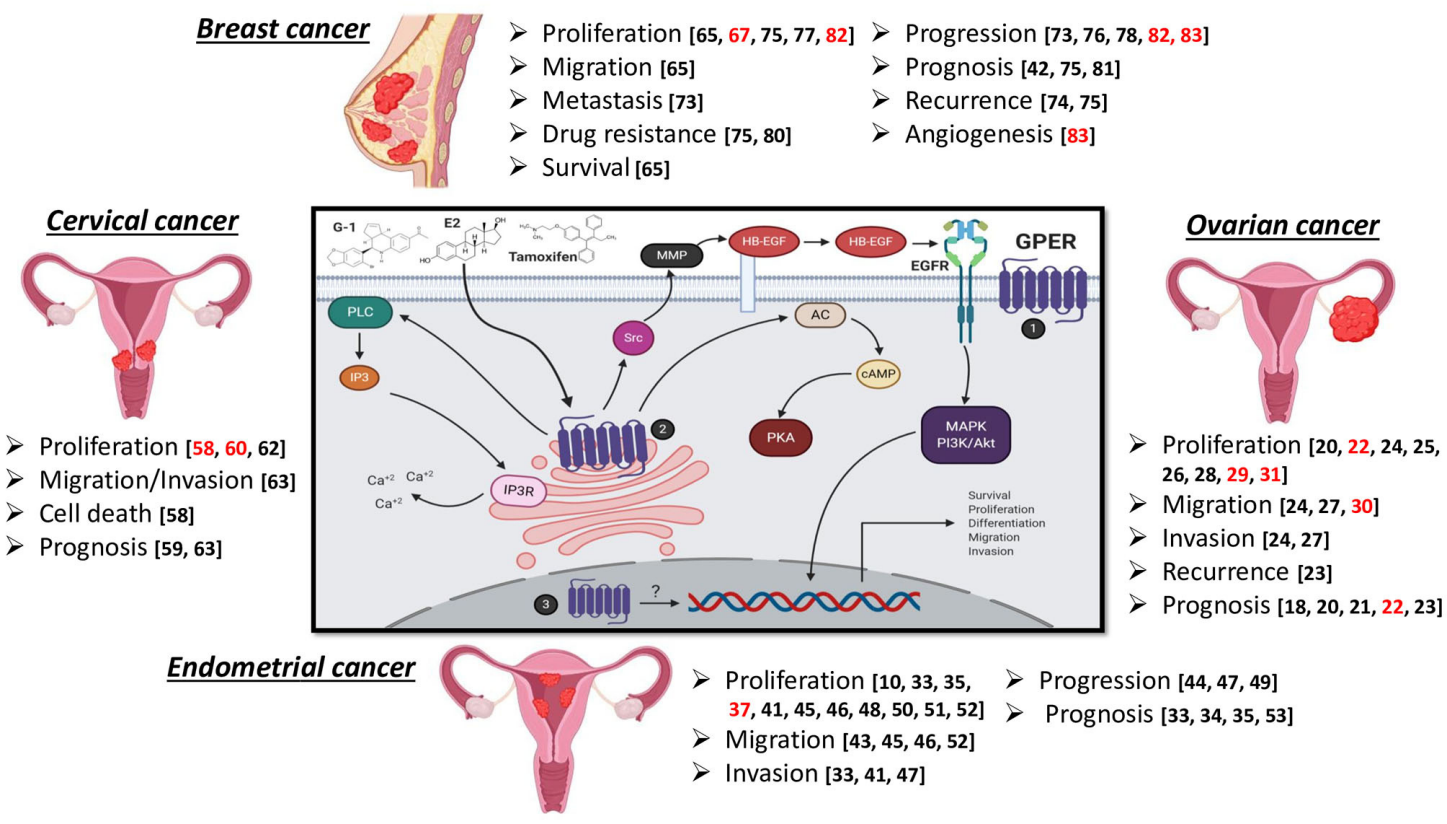
GPER signaling pathway and effect of its stimulation in female reproductive cancers. Subcellular location of GPER has been found at the plasma membrane (1), endoplasmic reticulum (2), and the nucleus (3). Black references refer to promotion of disease progression, such as cell proliferation, survival, migration, invasion, metastasis, poor prognosis, drug resistance, and increased recurrence, while the references in red suggest an anti-tumor role. Created with BioRender.

GPER/GPR30 has been classically described as a non-genomic receptor, which exerts rapid signaling actions. GPER belongs to the GPCR family, and it is canonically classified as a membrane-bound protein. Still, there is controversy over the fact that its expression has been found not only at the plasma membrane but also at the endoplasmic reticulum and the nucleus in some cases. GPER activation leads to cAMP production and PKA activation. Furthermore, such activation promotes the mobilization of calcium from the endoplasmic reticulum through PLC. In addition, it activates Src proteins and promotes the activation of MMP-2/9, resulting in EGFR transactivation. It is also able to consecutively activate MAPK and PI3K/Akt to promote the expression of several genes associated with cell survival, proliferation, differentiation, migration, and invasion (Figure 1) (11, 15, 16).

### GPER Activation in Female Reproductive Cancers

Numerous studies have shown that GPER activation has a positive modulating effect on the molecular mechanisms that determine carcinogenesis. This protumor action has been reported in different female reproductive cancers. However, some reports in the literature suggest that GPER activation has an antitumor role (Figure 1). This review focuses on varied findings regarding the role of GPER in female reproductive cancers.

### Ovarian Cancer

Ovarian cancer tumors are classified according to their histopathology and biological behavior, dividing them into epithelial, germ cell, and sexual cord stromal tumors (17).

In ovarian tissue samples, GPER is broadly expressed in high-risk ovarian cancer, associated with lower 5-year survival rates (18). Also, it has been found that this receptor is expressed in both benign and malignant tumors, and nearly one-third of malignant tumors overexpressed mRNA of GPER (19). Besides, its co-expression, along with EGFR, was associated with more reduced progression-free survival in ovarian cancer patients (20). Furthermore, it was shown that GPER is overexpressed in granulosa cell tumors, predicting a poor outcome in newly diagnosed patients (21).

On the opposite side, low expression of GPER in ovarian cancer tissue samples compared with benign and low malignant potential tumors was shown. A decrease in GPER expression was also reported during disease progression, which correlated with disease-free survival (22).

Regarding the receptor GPER localization, it was overexpressed in the nucleus and cytoplasm of serous and mucinous ovarian adenocarcinoma biopsies; its expression was higher in both advanced stages and patients with recurrence. Besides, its nuclear expression was predictive of poor overall survival and poor 5-year progression-free survival. GPER is also expressed in SKOV-3, OVCAR-3, and OVCAR5 cell lines and is abundantly co-expressed with ERα and ERβ in the SKOV3 cell line (22–24).

Studies in cell line models have been performed to determine the effect of GPER stimulation in ovarian cancer, and some of these studies have concluded that GPER activation promotes pro-oncogenic activities. For example, in the BG-1 cell line, G-1 induced the expression of pS2, cyclin A, cyclin D1, and cyclin E, while E2 promoted the expression of c-Fos and PR. Also, E2 and G-1 promoted cellular proliferation through pERK1/2 (25).

In OVCAR5 cells, E2 and G-1 agonists promote cell proliferation, increase the number of cells in the S phase of the cell cycle, increase the level of cyclin D1 and c-Fos, and reduce the level of active caspase 3 (26). Another study showed that both E2 and G-1 induce the migration and invasion in OVCAR5 cells, as well as the expression of MMP-9 and its proteolytic activity (27).

In the SKOV3 cell line, it was determined that GPER promotes cell proliferation by upregulation of c-Fos and cyclin D1 in a ligand-independent manner. Besides, it promoted migration and invasion, as well as MMP-2 and MMP-9 expression and their proteolytic activities (24).

Bisphenol A and tetrabromobisphenol A, two exogenous GPER ligands, have been reported to stimulate proliferation of OVCAR-3 and KGN cells (28). In the Caov-3 cell line, G-1 promotes this same cellular event through activation of the GPER/EGFR/Akt signaling pathway (20).

Some other studies conducted in cellular models have determined that the GPER stimulation is the opposite: the promotion of anticancer effects. For example, in SKOV-3 and OVCAR-3 cell lines, the specific stimulation of GPER with G-1 inhibited cell proliferation by induction of cell-cycle arrest in the sub-G1 phase as well as apoptosis (22).

Another study proved that G-1 alters morphology, decreases viability, suppresses proliferation, and induces apoptosis in IGROV-1 and SKOV-3 cell lines. Among the mechanisms involved in these processes, increased expression of the cell-cycle inhibitor P21CIP1 was found, as well as DNA fragmentation, decrease in the levels of the anti-apoptotic protein BCL-2, significant increase in cleaved PARP and fodrin, and microtubule assembly blockage during cell-cycle progression. In the SKOV-3 cell line, an increase was found in caspase 3/7 activity, while no significant changes were found in IGROV-1 cells. In addition, G-1 did not increase the expression of apoptosis-inducing factor (AIF) in IGROV-1 cells, but its translocation from the mitochondria to the nuclear area was observed (29).

E2 was reported to reduce the migration of OVCAR-3, SKOV3-IP, HEY, and TOV-112D cell lines by interfering with the expression of uPAR (30).

In KGN cells, G-1 alters cell morphology and suppresses proliferation independently of GPER, by increasing caspase 3/7 activity and arresting cell cycle in the G2/M phase (31).

### Endometrial Cancer

Endometrial cancer (EC) is classified in types I and II, according to the histological grade and myometrial invasion. Overweight, obesity, familiar history of EC, diabetes, nulliparity, and race are between the leading risk factor for the development of EC; however, one of the most important is the continuous exposure to endogenous or exogenous estrogenic stimulation, which is considered to be the cause of ~90% of the cases and represents the majority of type I EC, while type II is not related to estrogen (32).

Tamoxifen, a genomic estrogen receptor (ER) antagonist used in the treatment of breast cancer, is also considered an important risk factor for the development of EC. Endometrial tumors are heterogeneous with respect to ER expression. The fact that tamoxifen can act as GPER agonist was recently discovered (10); thus, the likely protumor effect is considered, which is the reason why performing further comprehensive research about the effects of GPER stimulation on the carcinogenesis of endometrial cancer, as well as the underlying mechanisms, is imperative.

GPER is overexpressed in EC biopsies and correlates with advanced stages of the disease, as well as high histopathological grade, aggressive subtypes, deep myometrial invasion, and poor overall survival, and it is similarly expressed between types I and II EC, with no differences between menopausal status. In addition, it is expressed in the luminal and basal surface of the EC epithelium. Furthermore, GPER was expressed higher in patients receiving tamoxifen treatments (33–36). Contrarily, a loss in GPER expression in EC tumors compared to the normal endometrium was observed (37), and this was correlated with a high FIGO stage, high histological grade, non-endometrioid histology, aneuploidy, low ERα expression, and disease progression (38). On the other hand, in cell lines derived from endometrial tumors, the expression of GPER is also varied, in RL95-2 it is high, in HEC-1A moderate, and in HEC-1B absent (37). Regarding its location, GPER was found at the cytoplasm and plasma membrane of the KLE and RL95-2 cell lines, and in normal endometrium, the location was predominantly intracellular (33, 35).

GPER mRNA expression has also been reported in the normal endometrium, finding higher levels in proliferative phases compared to secretory ones within the menstrual cycle. Additionally, epithelial cells contained a higher expression of GPER mRNA than stroma cells. At the protein level, the expression is present at both endometrial and decidua tissue in glandular, luminal, and stromal cells (39).

It has also been described that E2, G-1, OHT, IGF-1, and insulin favor the increased expression of GPER at mRNA and protein levels (35, 40–42). Several studies have reported that direct stimulation with E2, G-1, and OHT increases the carcinogenic characteristics of EC cell lines, such as proliferation, migration, and invasion, involving mechanisms such as activation of signaling pathways like MAPK and PI3K, calcium influx via Cav1.3 and DAKα, phosphorylation of FAK, and increased production of MMP-2/9, c-Fos, and cyclin D1 (33, 35, 40, 41, 43–50). However, only one report showed the inhibition of proliferation in RL95-2 and HEC-1A cell lines stimulated with G-1 in a dose-dependent manner (37). Also, miR-195 controls GPER expression *in vitro* in AN3-CA and HEC-1A cells (44).

Other signaling pathways have been found to interact with GPER to induce a plethora of effects in EC cell lines. IGF-1 triggers the expression of cyclin D1 and CTGF, promoting proliferation and migration of Ishikawa cells (42). Gankyrin decreases PTEN activity, leading to the activation of the GPER/PI3K/Akt pathway due to the stimulation with E2, which promotes the expression of cyclin D1 and, consequently, RL95-2 cell proliferation. This finding supports the fact that gankyrin and PTEN are inversely correlated in endometrial cancer biopsies (51). Another study found that the stimulation of GPER promotes the activation of the EGFR/MAPK pathway and induces the expression and recruitment of Egr-1 to the promoter region of CTGF and cyclin D1 genes in Ishikawa cells and CAFs treated with E2, G-1, and OHT (52). GPER was highly expressed in endometrial cancer biopsies that exhibited insulin resistance and positively correlated with TET1, a protein that regulates hydroxymethylation of DNA at the GPER promoter region, increasing its expression via the PI3K/Akt signaling pathway (40). The autocrine motility factor (AMF) interacts with GPER activating the PI3K/Akt pathway, promoting proliferation, and regulating apoptosis in SPEC-2 cells. In EC tissue, a high expression of both GPER and AMF has been correlated with poor prognosis in patients (53).

The capacity of GPER stimulation to induce tumors was studied in animal models. One of these studies found that the receptor activation promotes the ability of the RL95-2 cell line to produce a solid tumor in a xenograft model of athymic mice (47). Besides, GPER blockade inhibited growth tumor in an athymic mouse model based in HEC-1A cell line xenograft (49).

### Cervical Cancer

High-risk human papillomavirus (HPV) is necessary for the development of cervical cancer (CC) but requires the participation of other factors as estrogen signaling (54). Its signaling discovered the effects of estrogen on cervical carcinogenesis through nuclear receptors, mainly ERα, in murine models (55, 56). Besides, a study reported that both ERα and ERβ increase their expression as the disease progresses from cervical intraepithelial neoplasia (CIN) to CC (57). Another study showed the immunopositivity to GPER in CC, and its expression in CIN and normal cervical epithelium. A predominant localization at the cytoplasm and nucleus of the cervical tissues was found, and GPER expression in CIN samples was statistically significantly lower than in normal epithelium and CC samples (58). The expression of this receptor was highly found in CC tissue samples, and its localization was reported to be cytoplasmic and at the plasma membrane. In addition, a positive correlation between cytoplasmic GPER expression and the tumor suppressor proteins p16 and p53 was found; its expression was associated with a favorable prognosis of the disease (59).

GPER expression has been detected in CC-derived cell lines HeLa, SiHa, C-33A, and Caski, and its stimulation with G-1 reduces the viability of HeLa and SiHa cell lines through the activation of ERK1/2 by dysregulating cyclin B and inducing cell-cycle arrest in G2/M phase (60). In another report, stimulation of GPER with G-1 was shown to inhibit cell proliferation of HeLa, SiHa, and C-33A cell lines by inducing processes such as apoptosis, necrosis, and senescence (58). Besides, it was demonstrated in a transduced non-tumorigenic keratinocyte model that E6 and E7 oncogenes from HPV 16 and HPV 18 increase GPER expression at both mRNA and protein levels and that E7 oncogene modulates GPER localization in the nucleus (61).

Mono-ethylhexyl phthalate (MEHP), an environmental estrogenic chemical, can promote the proliferation of HeLa and SiHa cervical cancer cells through the GPER/PI3K/Akt pathway but have no effect over invasion and expression of MMPs (62).

A high GPER expression has been reported in cervical adenocarcinoma cell lines HeLa229, OMC4, HCA1, CAC-1, and TMCC1 and in tissue samples of cervical adenocarcinoma collected from patients. E2 and G-1 stimulation increases claudin-1 expression, contributing to malignant potential by increasing proliferation of CAC-1 and HCA1 cell lines through MAPK/ERK and PI3K/Akt. Furthermore, it was also observed that knocking out for claudin-1 expression in the TMCC1 cell line reduces its capability for proliferation, invasion, and migration. In a mouse xenograft model, tumors derived from GPER-knockout cells were smaller and grew slower than those derived from the control cells. Finally, this report found a positive correlation between GPER and claudin-1 co-expression and lower overall survival in cervical adenocarcinoma patients (63).

### Breast Cancer

Prolonged exposure to estrogen is the leading risk factor for breast cancer progression. In breast cancer cells, E2 enhances the stimulatory effects by binding to estrogen nuclear receptors, which regulate the expression of genes that contribute to the proliferation, migration, and survival of cancer cells (64, 65).

The aggressiveness of breast cancer tumors is related to the presence or absence of estrogen receptors, being classified into positive (RE+) and negative (RE–) tumors for the estrogen receptor (66), with RE– being intrinsically more aggressive due to the lack of effectiveness of treatments based on tamoxifen and aromatase inhibitors (67).

There is controversy about the location of GPER. Although GPER belongs to a family of surface receptors, which conventionally mediate transmembrane signaling of cell membrane-impermeable ligands, numerous studies have shown that GPER is detectable both in the plasma membrane and intracellularly in breast cancer cells (68–70).

As in other neoplasms, in breast cancer, the function of GPER is not clear yet. GPER has been proposed as a mediator of the estrogen action in breast malignancies, regulating critical biological responses to estrogens, such as changes in gene transcription, proliferation, and cell migration within the tumor microenvironment (70–72). Besides, it has been associated with increased tumor size, high risk of metastatic disease, recurrence, and reduced survival rates in patients with breast cancer (10, 73–75) favoring disease progression (76).

The proliferative effect generated by GPER has not only been studied in breast tumor tissues. In explants of non-tumor breast tissues, it was observed that G-1, the selective agonist of GPER, was able to stimulate the proliferation of tissues in culture. In contrast, G36, a GPER antagonist, blocked G-1-induced proliferation in non-cancerous human breast tissues (77).

An essential event for the spread and progression of carcinomas is the mesenchymal–epithelial transition (MET) in which GPER participates directly. This was demonstrated in breast cancer cell lines incubated with G15, a GPER antagonist. It was observed that G15 prevents breast cancer cells from undergoing mesenchymal–epithelial transition via GPER inhibition, and a synergistic effect was observed when incubating cells with doxorubicin and G15, causing increased sensitivity to this drug by the breast cancer cell lines (78).

GPER plays an important role in the development of resistance to treatment in breast cancer because RE antagonists such as tamoxifen and fulvestrant act as GPER agonists, stimulating proliferation and cell growth. Cellular treatment with tamoxifen for prolonged periods increases the overregulation of GPER stimulated by E2 and its relocation of the endoplasmic reticulum to the cell membrane (75).

Tamoxifen acts as a growth factor due to its ability to transactivate EGFR via GPER; this is the mechanism by which the MCF-7 cell line develops resistance to endocrine therapy (79). A significant correlation between GPER expression and the expression of EGFR and HER-2 has been observed. GPER-positive tumors are often less responsive to tamoxifen therapy due to the resistance generated by GPER (75).

Cytoplasmic GPER enhances the GPER/cAMP/PKA signaling pathway in breast cancer-associated fibroblasts, generating high tumor metabolic activity and resistance to tamoxifen, herceptin-2, and epirubicin treatment. It also promotes the transfer of high levels of energy between stromal cells and cancer cells (80).

For these and other studies, GPER has been proposed as a biomarker predictive of biologically aggressive phenotypes associating it with adverse results and poor survival of breast cancer patients. That is why GPER could be a therapeutic target (42, 81).

Controversially, some studies assign an antitumor function to the GPER. It was observed that estrogens significantly suppress breast cancer growth, inducing cell-cycle arrest in the G1 phase during hypoxia through GPER activation. This is due to low expression levels of ERα and the enhanced activation of GPER by estrogen. In addition, a positive correlation has been determined between GPER expression and adverse clinical outcomes in patients with breast and ovarian cancer (82).

Another study showed that GPER activation could significantly inhibit the cellular proliferation of ER- breast cancer. They observed that, in cultured breast cancer cells, treatment with G-1 decreased the expression of cyclin B, induced the arrest of the cell cycle in the G2/M phase, and caused apoptosis related to mitochondria, concluding that the activation of GPER can inhibit proliferation *in vitro* and *in vivo* through the generation of reactive oxygen species (ROS), apoptosis through the caspase pathway, and the decrease in cyclin B expression (67).

Additionally, in cell lines and in murine models of triple-negative breast cancer (TNBC), it has been observed that the activation of GPER by its G-1 agonist significantly inhibits the expression of IL-6 and VEGF-A and is also capable of suppressing the angiogenesis and progression of TNBC (83).

The effects of G-1 on proliferation and survival are highly controversial. Different studies suggest that the concentration of G-1 in the tumor microenvironment defines its function; some reports have shown that G-1 stimulates proliferation of breast cancer cells in a dose-dependent manner, in the range of 10 nM to 1 μM (52, 65, 77, 84), while micromolar levels potentially suppress the growth of breast cancer cells (65, 67, 85, 86).

There is evidence that G-1 can suppress the proliferation of breast cancer cells and induce cell apoptosis independently of GPER. However, the intracellular target and the mechanisms of inhibition of cell proliferation and induction of tumor cell apoptosis carried out by G-1 are unknown, demonstrating the need to deepen the study about the existence of other receptors for G-1 and the functions they exert after forming the receptor–ligand complex (31).

The expression pattern of GPER and its subcellular location is still a debate because it has been found at different sites within the cell. Using murine knockout models for GPER, it has been shown that its overexpression and its location in the plasma membrane are important events for breast cancer progression (87), while the absence of GPER in the plasma membrane has an excellent long-term prognosis in patients with ERalpha + breast cancer treated with tamoxifen (88, 89).

On the other hand, the expression of cytoplasmic GPER in breast carcinomas is associated with low tumor stages, better histological differentiation, and a better overall clinical outcome; the expression of nuclear GPER is associated with less favorable tumor properties. This indicates that GPER can have different cellular functions depending on its subcellular location and influence the development and prognosis of the disease (90, 91).

#### GPER Modulates the Intracellular Calcium Flow in Breast Cancer

The positive and negative effects that GPER has on the proliferation of various cell lines have been attributed to its modulating effect on intracellular calcium flow. GPER is capable of coupling to different Ca^2+^ channels such as IP3R (inositol triphosphate receptors) in ER-positive MCF-7 cells and ryanodine receptors (RyR) in ER-negative SKBr3 cells. Sustained abnormal increases in intracellular levels of Ca^2+^ may lead to inhibition of proliferation and induce apoptosis. Partial inhibition of the plasma membrane Ca^2+^-ATPase in MCF-7 cells causes a moderate increase in intracellular levels of Ca^2+^, leading to inhibition of proliferation altering the cell-cycle kinetics (92).

### Relationship Between GPER and Immune Response

Estrogen is extensively related to the modulation of cellular responses of the immune system and is largely studied within the pathology of inflammatory and autoimmune diseases context, mainly due to its actions through ERα/β (93). However, its role in cancer immunology is poorly understood.

GPER has been found to be expressed in adaptive and innate immune response cells such as circulating B and T lymphocytes and monocytes (94), macrophages (95, 96), neutrophils (97–99), eosinophils (100), and dendritic cells (98).

GPER is necessary for the apoptosis of double-positive thymocytes and contributes to thymic atrophy (101) as well as the survival of naïve T cells in mice (102). GPER-specific stimulation with G-1 promoted the production of IL-10 in CD4^+^ T cells cultured *ex vivo*, under Th17-polarizing conditions, similar to several autoimmune diseases via MAPK/ERK (103) and induced FOXP3 expression in regulatory T cells (104).

G-1 is capable of inhibiting the production of IL-6, TNF-α, IL-12 (p40), and CCL5 in human macrophages treated with LPS. Besides, G-1 reduced disease severity in an EAE multiple-sclerosis model (105).

In human neutrophils obtained from healthy donors, GPER activation increased the respiratory burst, cell viability, and expression of CD11b and CD62L, two markers of neutrophil activation. Likewise, G-1 promoted the expression of *IL1B, CXCL2, COX2, SOCS3, GCSF*, and *IL1RA* genes and increased the production of CXCL8 protein. These effects occurred through the cAMP/PKA/CREB, p38 MAPK, and ERK signaling pathways (98).

G-1 magnified the effect of eotaxin on eosinophil chemotaxis and inhibited spontaneous apoptosis of eosinophils by reducing caspase-3 activity, but it had the opposite effect on eosinophils previously stimulated with IL-5, a cytokine that promotes their survival in eosinophilic inflammation, inducing apoptosis by increasing caspase-3 activity. Nevertheless, GPER activation had no effect on eosinophil degranulation (100).

All these data suggest that estrogen signaling through GPER may have a modulatory effect on the immune system by decreasing important features for inflammation. This could lead to a protective role for autoimmune diseases, as occurs in multiple sclerosis studies; and therefore, immune system modulation by GPER stimulation could be investigated as a potential clinical target in inflammatory diseases.

#### What Effects Does GPER Have on the Expression of Cytokines Found in Cancer?

Little is known about the role that GPER activation could play over the induction of immune system response or other important components, as cytokine expression, on cancer. Here we reviewed the information published to date.

In endometrial cancer, GPER stimulation with both E2 and G-1 increased IL-6 expression through the MAPK pathway in KLE and RL95-2 cell lines (33). In addition, GM-CSF, VEGF, and IL-8 levels are increased and associated with high GPER expression in primary cultures from endometrial cancer tumors (106).

It has been observed that GPER activation reduces TNFα-induced IL-6 expression in the SKBR3 cell line and reduces IL-6 and VEGF-A levels in triple-negative breast cancer cell lines MDA-MB-231 and BT-549 through inhibition of NF-κB transcriptional activity (83, 107). Likewise, E2 prevented the action of TGF-β in the migration of MCF-7 and MDA-MB-231 breast cancer cell lines via GPER/ERK1/2, resulting in an inhibitory effect of Smad signaling (108).

Additionally, E2 and G-1 induced IL-1β expression in CAFs, while in MCF-7 and SKBR3 breast cancer cell lines they promoted IL1R1 expression, stimulating migration and invasion of breast cancer cells (109). Interestingly, it was observed that the lack of N-glycosylation in the N-terminal portion of GPER drives this receptor to the nucleus, becoming a transcription-like factor that promotes the expression of CTFG in CAFs and in the SKBR3 cell line. CTFG is a cytokine that increases MDA-MB-231 cell line migration capacity (110).

Tumor-promoting inflammation and immune system modulation by cancer cells are well-recognized as hallmarks for cancer progression (111). The evidence mentioned above suggests that GPER signaling is involved in the expression of cytokines by neoplastic cells, which are related to migration and angiogenesis. Nevertheless, there is a lack of information demonstrating the effect of GPER stimulation over the immune system's cells, as well as their interaction in the tumor microenvironment. Therefore, the elucidation of these mechanisms is necessary for a better understanding of the effect of GPER in carcinogenesis and tumor progression, making the estrogen signaling a potential therapeutic target in female reproductive cancers.

## Conclusion

Exposure to estrogen for long periods represents the main factor in the development of several cancers, including ovary, endometrial, cervical, and breast cancer. The main effects of estrogen through their receptors are related to cell survival, growth, and proliferation. Nevertheless, little is known about the effect of estrogen signaling through the G protein-coupled estrogen receptor in female reproductive cancers, as well as in the modulation of the immune system in these cancers. In general, it has been related to protumor processes such as increased cell survival, proliferation, migration, metastasis, and tumor growth, as well as the production of cytokines, which promote these effects. However, it is important to mention that there are some reports that show complete opposite results than the aforementioned studies, suggesting a possible antitumor role. This makes it difficult to propose a specific conclusion and creates the need for additional thorough research in this field.

## Author Contributions

CH-S, JV-P, and AP-S planned, wrote, and contributed to the critical review of the manuscript. In addition to these activities, AP-S directed this work. All authors contributed to the article and approved the submitted version.

## Conflict of Interest

The authors declare that the research was conducted in the absence of any commercial or financial relationships that could be construed as a potential conflict of interest.
